# Idiopathic multicentric Castleman disease with Sjögren’s syndrome and secondary membranous nephropathy: a case report and review of the literature

**DOI:** 10.1186/s12882-020-02191-z

**Published:** 2020-12-04

**Authors:** Yuejuan Pan, Zhuan Cui, Song Wang, Danxia Zheng, Zhenling Deng, Xinyu Tian, Hongxia Guo, Wenhan Bao, Sijia Zhou, Yue Wang

**Affiliations:** Department of Nephrology, Peking University Third Hospital, 49 Huayuanbei Road, Beijing, 100191 PR China

**Keywords:** Idiopathic Castleman disease, Membranous nephropathy, Sjögren’s syndrome, Tocilizumab, Cyclophosphamide

## Abstract

**Background:**

Idiopathic multicentric Castleman disease (iMCD) is an uncommon lymphoproliferative disorder and lacks treatment consensus. Herein, we report a case of iMCD complicated with Sjögren’s syndrome (SS) and secondary membranous nephropathy (SMN).

**Case presentation:**

A 45-year-old female with dry mouth for 3 months and anasarca and proteinuria for 2 months was admitted. She also experienced chest tightness, wheezing, fever, weight loss, moderate proteinuria and hypoalbuminemia. A computed tomography (CT) scan revealed a tissue mass in the thymus area and enlarged multiple lymph nodes. Her symptoms did not improve after resection of the thymus mass. The pathological findings were “reactive hyperplasia of the mediastinal lymph nodes and thymic hyperplasia”. Lymph node biopsy findings confirmed iMCD with human herpes virus-8 (HHV-8) negativity. Based on anti-nuclear antibody (ANA) 1:320, anti-SSA and anti-SSB antibody positivity, salivary flow less than 0.1 ml/min and lip biopsy with focal lymphocytic sialadenitis, SS was diagnosed. Kidney biopsy showed secondary membranous nephropathy with endocapillary cell proliferation and infiltration of plasma cells and lymphocytes in the tubulointerstitium. Serum interleukin-6 (IL-6) levels were significantly increased, and therapy with tocilizumab (anti-IL-6 receptor antibody) worked well. The combination of cyclophosphamide (CyS) with methylprednisolone (MP) maintained satisfactory remission.

**Conclusions:**

Our case of iMCD with SS and SMN is rare. There is a need for increased awareness of the disease to avoid unnecessary procedures and misdiagnoses. IL-6 was extremely high, and there was a rapid response to anti-IL-6 receptor agents. The combination of CyS with MP maintained complete remission.

## Background

Benjamin Castleman originally described Castleman disease (CD) in the 1950s [1]. CD was first reported in a series of patients with few or no symptoms but solitary mediastinal lymph node hyperplasia [2]. The etiology of CD is unknown, but the recognized central factors include lymph node hyperplasia with polyclonal B lymphocyte expansion and cytokine storms (IL-6 and vascular endothelial growth factor VEGF) [3].

Clinically, CD is divided into two subtypes: unicentric CD (UCD) and multicentric CD (MCD). Histologically, CD is classified into three distinct entities: hyaline vascular CD, plasma cell CD and mixed-type CD, in which the hyaline vasculature correlates with UCD. In contrast, plasma cell CD is more related to MCD [4]. MCD can be further subdivided into iMCD (HHV-8 and human immunodeficiency virus-negative MCD) and HHV-8 or HIV-associated MCD [5]. A subgroup of MCD patients have POEMS (polyneuropathy, organomegaly, endocrinopathy, M protein) or TAFRO (thrombocytopenia, anasarca, fever, reticulin fibrosis, organomegaly) syndrome [6].

The rarity of MCD makes it a challenge to diagnose, treat and follow-up. Twenty-five percent of new CD cases in the United States were reported to be iMCD, with a median age at diagnosis of approximately 50–65 years [7, 8]. Additionally, a retrospective study in 2014 showed that the 5-year survival rate of MCD was approximately 28% less than that of UCD [9]. A systemic literature review by Sitenga et al. [10] involving 7 studies found 5-year survival rates of nearly 96.4% for siltuximab (anti-IL-6 chimeric monoclonal antibody) therapy.

Here, we report a case of a patient with iMCD complicated with SS and SMN.

## Case presentation

A 45-year-old Chinese female herdsman was admitted to our hospital with dry mouth for 3 months and anasarca and proteinuria for 2 months. She also had chest tightness, wheezing and weight loss of 10 kg within 3 months. CT revealed an abnormal tissue mass in the thymus area and multiple enlarged lymph nodes located in the mediastinum, subclavian, and bilateral underarm. Laboratory tests showed hypoalbuminemia (25.2 g/L), proteinuria (4.9 g/g creatinine) and normal renal function (eGFR 96 mL/min/1.73m^2^). She underwent anterior mediastinal mass and partial pericardial resection with the pathological findings “reactive hyperplasia of the mediastinal lymph nodes and thymic hyperplasia”. The symptoms did not improve after the operation. Her body temperature rose to 38.1 °C, with cough and sputum, and returned to normal after antibiotics. One month later, she had a fever again that did not subside after antibiotic treatment. On admission, her temperature was 37.4 °C, and her blood pressure was 116/70 mmHg. Superficial lymph nodes were palpable in the subclavian, bilateral underarm and groin. Edema of the bilateral lower extremities was present. Laboratory findings revealed normal white blood cell, red blood cell (RBC), and platelet counts and a serum creatinine level of 69 μmol/L; microscopic hematuria (RBC 11–30/HP), proteinuria (2.6 g/24 h), lowered serum albumin (31 g/L), high ESR (50 mm/h), high CRP (15.9 mg/L), high IgG (22.1 g/L, normal rangen 6.94–16.18 g/L), normal IgA and IgM and extremely elevated IL-6 (4601 pg/mL, normal range < 3 pg/mL) levels in serum were also detected. There was no monoclonal peak on immunoelectrophoresis for either the serum or urine. ANA was 1:320. Both anti-SSA and anti-SSB were positive. Serum lupus anticoagulant, anticardiolipin antibody, anti-phospholipase A2 receptor antibody and complement levels were normal. Hepatitis viruses A, B and C, HIV, EBV and cytomegalovirus were negative. Unstimulated salivary flow was 1.2 mL/15 min. Lip biopsy showed focal lymphocytic sialadenitis. Thoracic and abdominal ultrasound scans indicated polyserositis with pleural and pericardial effusions and ascites. Thoracentesis was performed but failed to draw any pleural effusion. Nonetheless, 700 mL ascites was drained from the abdominal cavity; the ascites fluid culture was sterile.

**Fig. 1:**
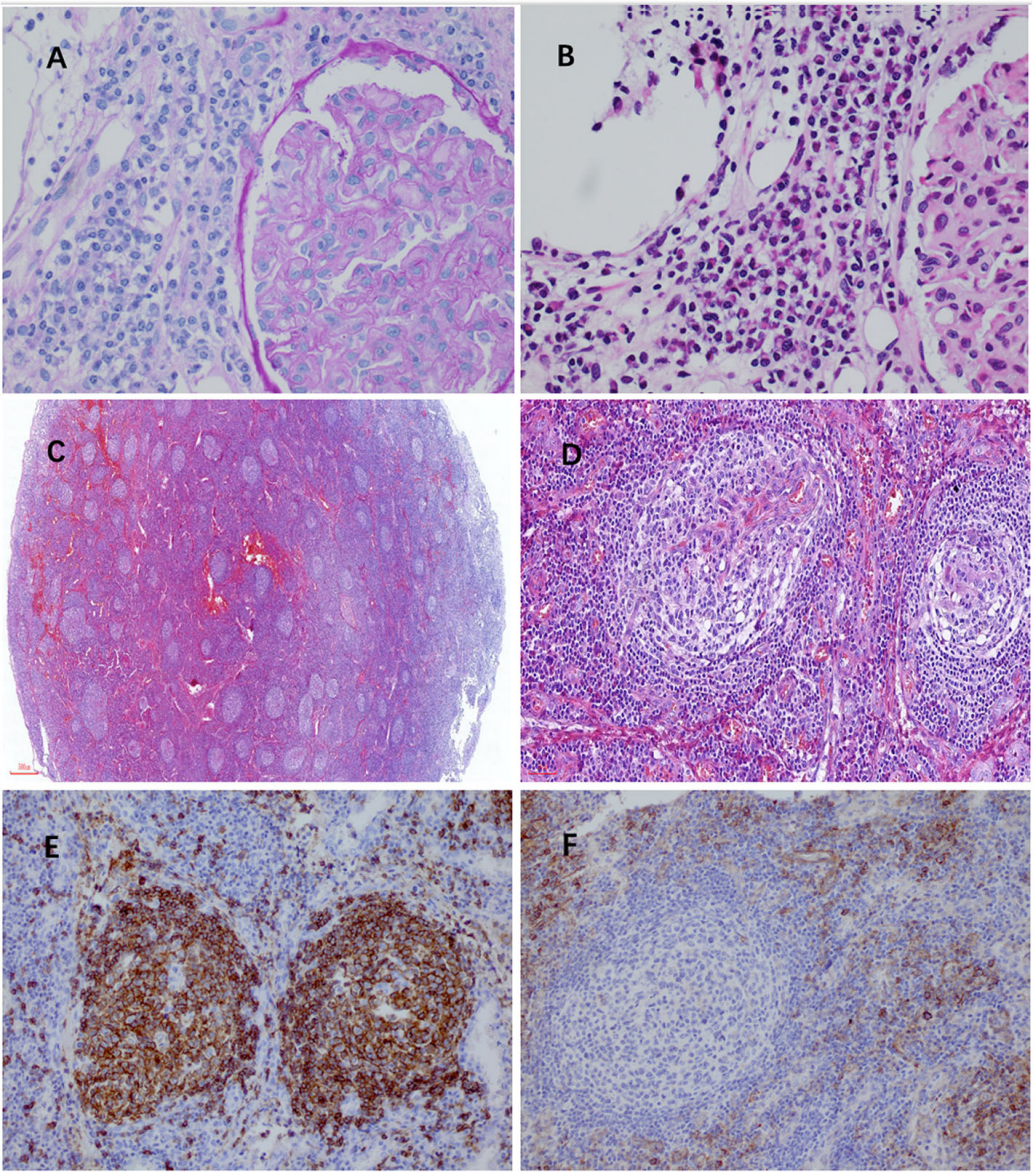
Histopathological findings on renal and inguinal lymph node biopsies. Renal (**a**-**b**) and lymph node biopsies (**c-f**) at the onset of disease. **a** Diffuse endocapillary and mesangial cell proliferation (PAS staining, × 400). **b** Lymphocyte and plasma cell infiltration in the tubulointerstitium (HE staining, × 400). **c** Lymphoid follicles were increased in number, and had hyperplastic germinal centers in the lymph nodes were detected (HE staining, × 40). **d** Shown are atrophic germinal centers, with small vessels reaching germinal centers, onion-skinning mantle zones (HE staining, × 200). **e** CD20^+^cells were mainly observed in follicular regions (× 200). **f** Plasmacytosis highlighted with CD138 staining in the interfollicular space is shown (× 200)

**Fig. 2:**
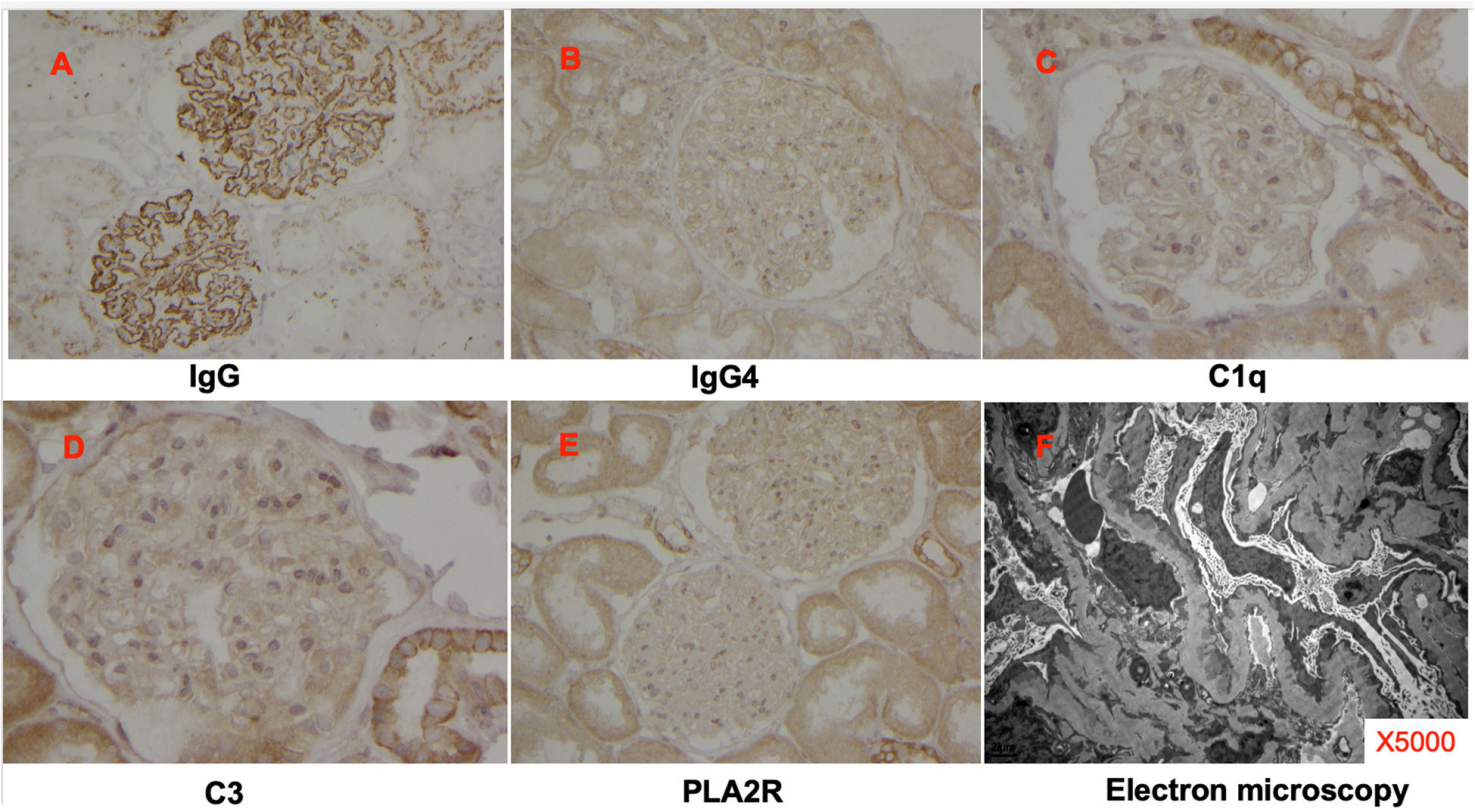
Immunohistochemistry and electron microscopy. A panel of antibodies were used as indicated (**a-e**). IgG was positive along the glomerular capillary walls and mesangium. IgG4, C1q, C3 and PLA2R were negative. Electron microscopy (**f**) showed expanded glomerular capillary walls, subepithelial electron-dense deposits and diffuse podocyte foot process fusion

Kidney biopsy findings were as follows. By light microscopy, diffuse global endocapillary and mesangial cell proliferation in 11 glomeruli with glomerular capillary wall expansion were detected, as was prominent lymphocyte and plasma cell infiltration in the tubulointerstitium (Fig. 1). According to immunofluorescence microscopy, fine granular deposition of IgG was observed along the glomerular capillary walls and in the mesangium. IgA, IgM, C1q, C3, IgG4, PLA2R and fibrinogen were negative (Fig. 2). Electron microscopy showed expanded glomerular capillary walls, subepithelial electron-dense deposits and diffuse podocyte foot process fusion (Fig. 2). The diagnosis was “secondary membranous nephropathy”.

Lymph node biopsy findings were as follows. Mediastinal and inguinal lymph node biopsy showed hyperplastic germinal centers as well as occasional atrophic germinal centers with small vessels reaching the germinal centers as well as interfollicular plasmacytosis. Immunohistochemistry revealed interfollicular plasmacytosis based on CD138 staining. CD20^+^B cells were mainly observed in follicular regions and occasionally in interfollicular regions. CD3^+^ T cells were mainly observed in interfollicular regions and sparsely in follicular regions. Ki-67, a cellular marker for proliferation, was highly expressed within the germinal centers. HHV-8 was negative. The diagnosis was “multicentric Castleman disease”.

### Clinical diagnosis

1. Idiopathic multicentric Castleman disease
2. Sjögren’s syndrome
3. Secondary membranous nephropathy

### Clinical course

**Fig. 3:**
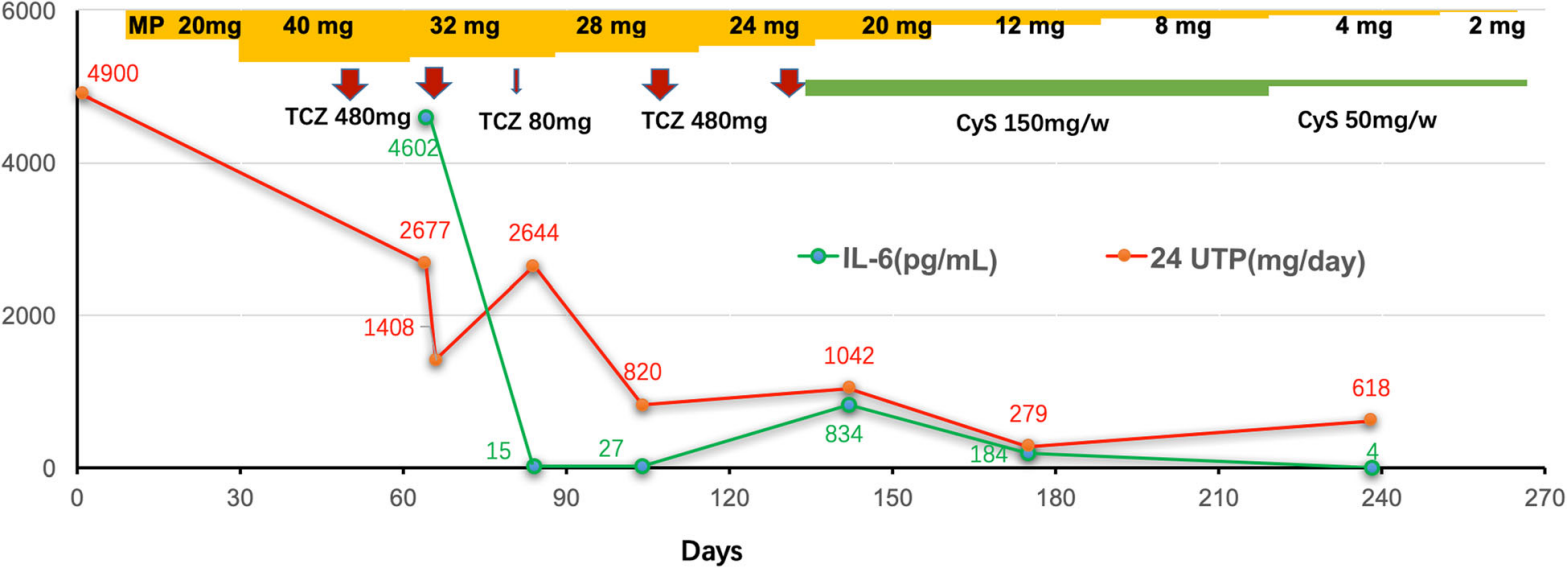
Treatment and prognosis. MP alone at the dose of 20 mg/d and 40 mg/d were given orally for the first and second month, but without efficacy. Anti-IL-6 receptor antibody (tocilizumab) treatment was started based on the result of elevated serum IL-6, at 4062 pg/mL, and given in a total of five sessions at 3–4-week interval. The serum IL-6 level returned to normal after the first dose of 480 mg, remained normal after the second dose of 480 mg, rebounded after tocilizumab was reduced to 80 mg at the third dose and returned to normal after the fourth and the fifth doses of 480 mg tocilizumab. As the serum IL-6 level decreased, the 24 h urinary protein level returned to the normal range, and edema, fever, polyserositis, serum albumin improved. CyS was added orally at the dose of 150 mg/week after tocilizumab was stopped and MP tapered. The IL-6 level, blood and urine tests and clinical manifestations all remained normal at the follow-up of 1 year after discharge with oral MP of 2 mg/day and CyS of 50 mg/week. IL: Interleukin; MP: methylprednisolone; 24-UTP: 24 h urine total protein; TCZ: tocilizumab; CyS: cyclophosphamide

The clinical course is illustrated in Fig. 3. Oral MP alone at a dose of 20 mg/d was ineffective after 1 month and was enhanced to 40 mg/d for another month without remission. Anti-IL-6 receptor antibody (tocilizumab) was started based on the result of elevated IL-6 (4062 pg/mL) in the serum and administered in a total of five sessions at 3–4-week intervals. Serum IL-6 level returned to normal after the first dose of 480 mg, remained normal after the second dose of 480 mg, rose after tocilizumab was reduced to 80 mg at the third dose and returned to normal after the fourth and the fifth 480 mg doses of tocilizumab. As the serum IL-6 level decreased, protein in the urine returned to the normal range, and her edema, fever, polyserositis, and serum albumin improved. CyS was added orally at a dose of 150 mg/week after tocilizumab was stopped and MP tapered. The IL-6 level, blood and urine tests and clinical manifestations all remained normal at the one-year follow-up after discharge with oral MP of 2 mg/day and CyS of 50 mg/week. Her symptoms of dry mouth were relieved, and her serum IgG returned to normal (12.8 g/L). However, but her ANA was still 1:320, and anti-SSA and anti-SSB were positive after 11 months of therapy.

## Discussion and conclusions

Although it was described 70 years ago, MCD remains a rare and life-threatening disease with poorly understood etiology, and its rarity and complexity make it a challenge for physicians and pathologists to reach an accurate diagnosis and provide reasonable treatment. Her symptoms, such as dry mouth, anasarca, chest tightness, wheezing and weight loss, were not specific, especially for surgeons who taken the mediastinal lymph node hyperplasia as thymoma and just performed resection of anterior mediastinal mass. It was unfortunate that biopsy of the enlarged lymph nodes was not performed before the surgery. Furthermore, the pathologist failed to distinguish or identify the pathological manifestations of MCD.

After CD is diagnosed, the type needs to be differentiated. In our case, lymph node biopsy revealed hyperplastic germinal centers as well as occasional atrophic germinal centers, with small vessels reaching the germinal centers and interfollicular plasmacytosis. CT showed multiple enlarged lymph nodes in the mediastinum, subclavian, and bilateral underarm. All these findings support the diagnosis of MCD. At the same time, HIV in the serum and HHV-8 in lymph node biopsy were both negative, and the diagnostic criteria of TAFRO syndrome or POEMS syndrome were not met.

SS may occur in isolation or in association with another systemic autoimmune disease, such as rheumatoid arthritis, systemic lupus erythematosus or scleroderma [11]. Numerous lymphocytes and plasma cells are the dominant infiltrating cells in the lymph nodes of patients with CD and in the salivary gland tissue of patients with primary SS. According to a report, IL-6 produced by local B lymphocytes can promote the synthesis of autoantibodies [12], and it has been reported that serum and saliva levels of IL-6 are higher in SS patients than in normal controls [13]. Coexistence of CD with SS has been published in two case reports [14, 15]. Biologic therapies against B lymphocytes (anti-CD20) such as rituximab have resulted in clinical remission in a case of MCD with SS [14]. However, a poor response to rituximab and siltuximab was reported for another case of MCD with TAFRO syndrome and SS [15]. At present, it is difficult to explain the causal relationship between MCD and SS. In our case, the symptoms and signs related to MCD and SS were relieved after 1 year of therapy, and serum IgG returned to normal. Nevertheless, ANA was still 1:320, and anti-SSA and anti-SSB were still positive.

Renal involvement with MCD has only been described in a limited number of small studies [13]. Glomerular pathologies mainly include amyloidosis [16, 17], thrombotic microangiopathy [18] and membranoproliferative glomerulonephritis [19]. Other renal pathological findings, such as mesangial proliferative glomerulonephritis, interstitial nephritis, membranous nephropathy, crescentic glomerulonephritis, minimal change disease and focal segmental glomerulosclerosis, are rare [13, 16–20]. Our patient developed SMN with endocapillary and mesangial cell proliferation and infiltration of plasma cells and lymphocytes in the tubulointerstitium.

MCD is characterized by a proinflammatory syndrome. IL-6 plays a vital role in the pathogenesis and clinical symptoms of patients with MCD [3, 21]. IL-6 is an important growth, differentiation and survival factor for both plasma cells and lymphocytes,leading to lymph node hyperplasia and elevated gammaglobulenemia [19]. IL-6 inhibits albumin production by hepatocyte, leading to hypoalbuminemia. IL-6 also induces VEGF secretion and increases vascular permeability, which in combination with hypoalbuminemia explains edema, ascites, and pleural effusion [3]. In our patient, overproduction of IL-6 accounted for a variety of clinical symptoms, including lymphadenopathy, hypoalbuminemia, elevated IgG and anasarca, among others.

A wide variety of treatments have been used to manage iMCD, including surgery, corticosteroids, rituximab or chemotherapy. Recently, monoclonal antibodies targeting the IL-6 signaling pathway have been approved for iMCD therapy [6, 22–24]. The latest report showed that iMCD and membranous nephropathy also respond to tocilizumab [17]. Our patient underwent thymus mass resection and initial corticosteroid therapy, which were not effective. The MCD consensus guidelines recommend an anti-IL-6 monoclonal antibody as the first-line therapy for patients with severe CD [6]. Tocilizumab was effective in our case. Indeed, polyserositis, proteinuria and fever were completely relieved after 3 doses. However, the serum IL-6 level rebounded after tocilizumab tapering. The antibody has a short half-life and cannot block large quantities of IL-6 [24]. Following the guidelines [6], we added oral CyS combined with MP, and remission was achieved.

In conclusion, Castleman disease is an uncommon lymphoproliferative disorder. There is a need for increased awareness of this disease to avoid unnecessary procedures and misdiagnoses, as was the case with this patient. The fact that patient with Castleman disease had additional disorders of secondary membranous nephropathy and Sjogren’s syndrome raises interesting questions about the pathogenesis of these disorders. Tocilizumab was effective in inducing remission, and the subsequent combination of CyS with MP maintained remission.

## Data Availability

All data and material are presented in this manuscript.

## Abbreviations

MCD: Idiopathic multicentric Castleman disease
CT: Computed tomography
SS: Sjögren’s syndrome
HHV-8: Human herpes virus-8
ANA: Anti-nuclear antibody
SSA: Sjögren’s syndrome-related antigen A
SSB: Sjögren’s syndrome-related antigen B
SMN: Secondary membranous nephropathy
IL-6: Interleukin-6
CyS: Cyclophosphamide
MP: Methylprednisolone
TCZ: Tocilizumab
POEMS: Polyneuropathy, organomegaly, endocrinopathy, M protein
TAFRO: Thrombocytopenia, anasarca, fever, reticulin fibrosis, organomegaly
RBC: Red blood cell
ESR: Erythrocyte sedimentation rate
CRP: C-reactive protein
HIV: Human immunodeficiency virus
EBV: Epstein-Barr virus
